# Chemoirradiation for Glioblastoma Multiforme: The National Cancer Institute Experience

**DOI:** 10.1371/journal.pone.0070745

**Published:** 2013-08-05

**Authors:** Jennifer Ho, John Ondos, Holly Ning, Sharon Smith, Teri Kreisl, Fabio Iwamoto, Joohee Sul, Lyndon Kim, Kate McNeil, Andra Krauze, Uma Shankavaram, Howard A. Fine, Kevin Camphausen

**Affiliations:** 1 Radiation Oncology Branch, National Cancer Institute, National Institutes of Health, Bethesda, Maryland, United States of America; 2 Neuro-Oncology Branch, National Cancer Institute, National Institutes of Health, Bethesda, Maryland, United States of America; 3 Radiation Management Associates, Bethesda, Maryland, United States of America; 4 Jefferson Medical College, Philadelphia, Pennsylvania, United States of America; University Hospital of Heidelberg, Germany

## Abstract

**Purpose:**

Standard treatment for glioblastoma (GBM) is surgery followed by radiation (RT) and temozolomide (TMZ). While there is variability in survival based on several established prognostic factors, the prognostic utility of other factors such as tumor size and location are not well established.

**Experimental Design:**

The charts of ninety two patients with GBM treated with RT at the National Cancer Institute (NCI) between 1998 and 2012 were retrospectively reviewed. Most patients received RT with concurrent and adjuvant TMZ. Topographic locations were classified using preoperative imaging. Gross tumor volumes were contoured using treatment planning systems utilizing both pre-operative and post-operative MR imaging.

**Results:**

At a median follow-up of 18.7 months, the median overall survival (OS) and progression-free survival (PFS) for all patients was 17.9 and 7.6 months. Patients with the smallest tumors had a median OS of 52.3 months compared to 16.3 months among patients with the largest tumors, *P = 0.006*. The patients who received bevacizumab after recurrence had a median OS of 23.3 months, compared to 16.3 months in patients who did not receive it, *P = 0.0284.* The median PFS and OS in patients with periventricular tumors was 5.7 and 17.5 months, versus 8.9 and 23.3 months in patients with non-periventricular tumors, *P = 0.005*.

**Conclusions:**

Survival in our cohort was comparable to the outcome of the defining EORTC-NCIC trial establishing the use of RT+TMZ. This study also identifies several potential prognostic factors that may be useful in stratifying patients.

## Introduction

Glioblastoma multiforme (GBM) is the most common primary central nervous system malignancy in adults, with approximately 14,000 newly diagnosed patients in the United States each year [1]. Despite multi-modality treatment, it remains one of the most aggressive tumors, with a median overall survival of only 14 months [2]. Nitrosoureas were the mainstay of adjuvant chemotherapy despite limited evidence of benefit [3] until 2005, when the European Organization for Research and Treatment of Cancer (EORTC) and the National Cancer Institute of Canada published a randomized prospective trial comparing surgery followed by either radiation therapy alone, or radiation therapy plus the addition of concurrent daily temozolomide and 6 months of adjuvant temozolomide (RT+TMZ) [2]. There was an improvement in median overall survival in the RT+TMZ group of 14.6 months compared to 12.1 months, and a 2-year overall survival of 27% compared to 11%. This regimen has been widely adapted as the standard of care since then.

Although the median survival is 14.6 months, there is a range of survival times when patients are placed in subsets using the recursive partitioning analysis (RPA) developed in 1993 (4). The RPA identified four risk groups based on several prognostic factors, with age (greater or less than 50 years) being the most significant determinant of survival, followed by performance status, mental status, neurological function, extent of resection, and radiotherapy dose. The prognostic significance of the RPA was validated in patients receiving RT+TMZ in 2006 [4] and simplified to include three distinct prognostic groups defined by age, performance status, extent of resection, and neurological function. The majority of recurrences occur locally with the predominance of failures occurring within the high dose radiation fields [5]. Consistent with this, in a recent dose escalation study of intensity-modulated RT (IMRT) using doses of 66 to 81 Gy, 18 out of 28 recurrent tumors had at least 80% of the recurrent volume within the 95% prescription isodose line [6].

Presented here are the experience and outcomes of patients treated at the National Cancer Institute in Bethesda, Maryland using the radiation and temozolomide protocol. Included are the radiographic recurrence patterns. Also reported are possible new prognostic factors, such as tumor location (periventricular versus non-periventricular) and primary tumor size, that may be useful in stratifying patients in future research protocols.

## Methods

### Patients

A total of 100 consecutive adult patients with histologically-confirmed (World Health Organization) grade IV GBM were treated with radiation therapy at the NCI in Bethesda, Maryland between 1998 and 2012 on a National Cancer Institute institutional review board approved protocol after given written informed consent. Eight patients were excluded from analysis: two patients who died during treatment, and six patients who did not complete radiation treatment because of clinical deterioration. Ninety two patients were included in the final analysis. All patients underwent surgery [gross total resection (GTR), sub-total resection (STR) or biopsy (Bx)], followed by external beam, involved field RT. Demographic factors including age, performance status, working status, and extent of resection prior to treatment were collected, and an RPA score was derived for all patients [4]. Other clinical parameters were collected for each patient, including gender, tumor location, chemotherapy regimens, time of last follow-up, and patient status at the last follow-up (alive or deceased).

### Tumor Location

The available pre-operative magnetic resonance imaging (MRI) sequences were reviewed. Patients were classified as “periventricular” if the contrast-enhancing lesion seen in T1-weighted MRI was in contact with the lateral ventricle, and all other patients were classified as “non-periventricular”.

### Treatment Plan

Patients were simulated and treated using a custom thermoplastic face mask for immobilization. A computerized tomography (CT) scan of the head and upper neck was obtained during simulation using a Philips Brilliant Big Bore CT scanner, and images were transferred to a Varian Eclipse treatment planning system. The MR images, including post-contrast T1 images, T2 images or fluid attenuated inversion recovery (FLAIR) images, were fused (co-registered) to the CT images, as previously described [7]. The majority of patients (91% of patients) received RT at a dose of 2 Gy given once daily, 5 days per week, for a total dose of 60 Gy over the course of 6 weeks. Gross tumor volumes (GTVs) were contoured using T2 or FLAIR MRI, and T1 MRI. The initial gross tumor volume, GTV1, was defined as the enhancing lesion and surrounding edema seen on T2 or FLAIR. The boost volume, GTV2, was defined as the contrast-enhancing lesion only, as seen on T1 MRI. The planning target volumes (PTVs) were volumetric expansions within the skull of the GTVs. PTV1 consisted of a 2 cm expansion of GTV1 and was treated to a total dose of 46 Gy in 23 fractions. The cone down volume, PTV2, consisted of a 2.5 cm expansion of GTV2, and was treated with seven additional 2 Gy fractions to a total dose of 60 Gy. The maximum dose limits to normal tissues and organs at risk were: 7 Gy to the lenses, 50 Gy to the retinae, 55 Gy to the optic nerves, 56 Gy to the optic chiasm, and 60 Gy to the brainstem. Target volumes were obtained using preexisting contoured 3D tumor volumes, and recorded in cubic centimeters (cc) using the “calculate volume” function in the treatment planning system.

### Chemotherapy

The majority of patients received RT with concurrent daily TMZ (90% of patients), followed by adjuvant monthly temozolomide (75%). Concurrent TMZ was prescribed at a dosage of 75 mg/m^2^/day, and adjuvant TMZ was prescribed at a dosage of 150 to 200 mg/m^2^/day for 5 days every 28 days for 6–12 cycles, unless the patient experienced disease progression, or treatment-related toxicity. Patients were treated with various therapies, including bevacizumab, after tumor progression, at the discretion of their treating physician. Anti-seizure medications and steroids were given as needed and doses were recorded at each treatment visit.

### Pattern of Failure

Conventional MRI was obtained at 1 month post-radiation and every 2–3 months thereafter. Response was defined using MacDonald criteria and more recently the RANO criteria [8]. For patients with MRI documented failures, the T1 MRI showing tumor recurrence was fused to the original CT used for treatment planning, and the contrast-enhancing lesion was delineated as the recurrent gross tumor volume (rGTV). The dosimetric location of the recurrence was determined by overlaying the dose distribution on the planning CT, and observing where the rGTV was located in relation to the 90% isodose line. Recurrence was defined as “central” when the entire tumor recurrence resided within the 90% isodose surface, as “marginal” if the tumor recurrence crossed the 90% isodose surface, and as “distant” was the tumor recurrence resided entirely outside of the 90% isodose surface [9].

### Statistical Analysis

The date of tumor progression was determined based on clinical symptoms and MRI-documented progression of disease. The date of death was determined based on clinic notes or using the internet-based Social Security Death Index. Progression-free survival and overall survival were measured from the date of diagnosis to the date of progression, death, or last follow-up. Time-to-event distributions were estimated using the Kaplan-Meier method and compared with the log-rank test. Cox regression was used for multivariate analysis.

## Results

### Patient Characteristics, Recurrence Pattern and Survival

**Figure 1:**
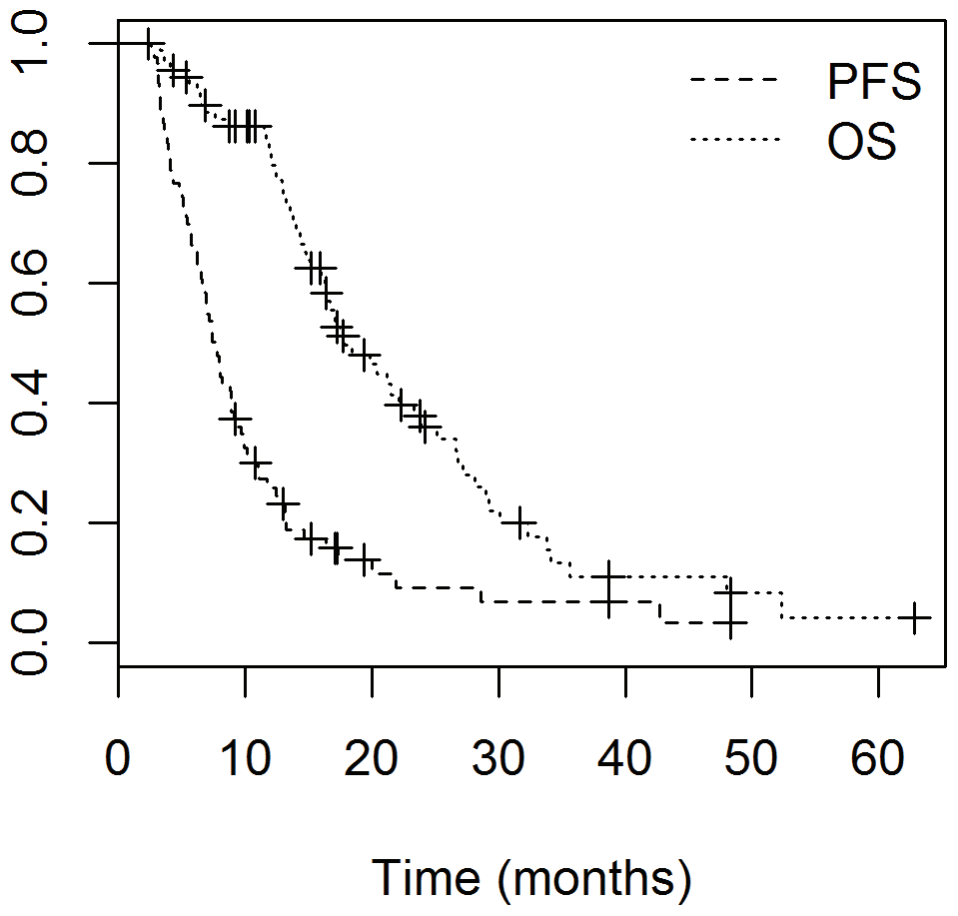
Kaplan-Meier analysis of progression-free survival (PFS) and overall survival (OS) in all patients. The median OS for all patients was 17.9 months (95% CI: 16.3–23.9) and the median PFS for all patients was 7.6 months (95% CI: 6.8–9.1).

The final patient cohort included 92 patients with GBM who were seen at the National Cancer Institute between July 1998 and January 2012. Patient characteristics for the entire cohort are listed in Table 1. The median patient age at diagnosis was 57 years (range: 31–79 years). Temozolomide was given concurrently to 83 patients, and in an adjuvant setting to 69 patients. Eighty patients completed the prescribed concurrent temozolomide. Of those patients who received adjuvant monthly temozolomide, 55 received temozolomide for ≥6 months. At a median follow-up of 18.7 months (range: 2.3–116.0 months), 70 patients had evidence of tumor progression, and 61 patients had died. The median OS for all patients was 17.9 months (95% CI: 16.3–23.9, Fig. 1) and the median PFS for all patients was 7.6 months (95% CI: 6.8–9.1, Fig. 1). Of the patients who had progressed, 56 had complete datasets including the radiation treatment plans and MRIs showing recurrence. Forty eight (86%) of the patients had a central recurrence; four (7%) had a marginal recurrence; and three (5%) had a distant recurrence. Twenty nine of the patients that recurred received bevacizumab, either as a mono-therapy or as part of combination therapy.

**Table 1:** Patient and Tumor Characteristics.

	Subcategory	n (%)
**Age (years)**		
	<50	20 (22%)
	≥50	72 (78%)
	median (range)	57 (31–79)
**Sex**		
	female	31 (34%)
	male	61 (66%)
**KPS**		
	90–100	76 (83%)
	70–80	13 (14%)
	<70	2 (2%)
**Working/Not Working**		
	W	77 (84%)
	NW	15 (16%)
**RPA**		
	3	19 (21%)
	4	49 (53%)
	5	24 (26%)
**Extent of Surgery**		
	Gross total resection	32 (35%)
	Subtotal resection	44 (48%)
	Biopsy only	16 (17%)
**Bevacizumab at 1st recurrence**		
	Yes	29 (41%)
	No	33 (47%)
**Concurrent temozolomide**		
	Yes	83 (90%)
	No	9 (10%)
**Adjuvant temozolomide**		
	Yes	69 (75%)
	No	18 (20%)
**Corticosteroids during RT**		
	Yes	67 (73%)
	No	25 (27%)
**Levetiracetam during RT**		
	Yes	56 (61%)
	No	36 (39%)
**Hemisphere**		
	right	38 (41%)
	left	53 (58%)
	both	1 (1%)
**Location**		
	Temporal	23 (25%)
	Parietal	18 (20%)
	Frontal	22 (24%)
	Occipital	2 (2%)
	Temporal-Parietal	5 (5%)
	Occipital-Parietal	6 (7%)
	Frontal-Temporal	6 (7%)
	Frontal-Parietal	4 (4%)
	Other	5 (5%)
**Failures**		
	central	49 (88%)
	marginal	4 (7%)
	distant	3 (5%)
**Tumor Location**		
	Periventricular	27 (29%)
	Nonperiventricular	59 (64%)
	Unsure	6 (7%)

Abbreviations: KPS, Karnofsky Performance Status; W, Working; NW, Not working; RPA, Recursive Partitioning Analysis; RT, Radiation Therapy. Patient demographics, treatment details, and characteristics of primary tumor before surgery/chemoirradiation.

### Prognostic Factors

#### Tumor size

**Figure 2:**
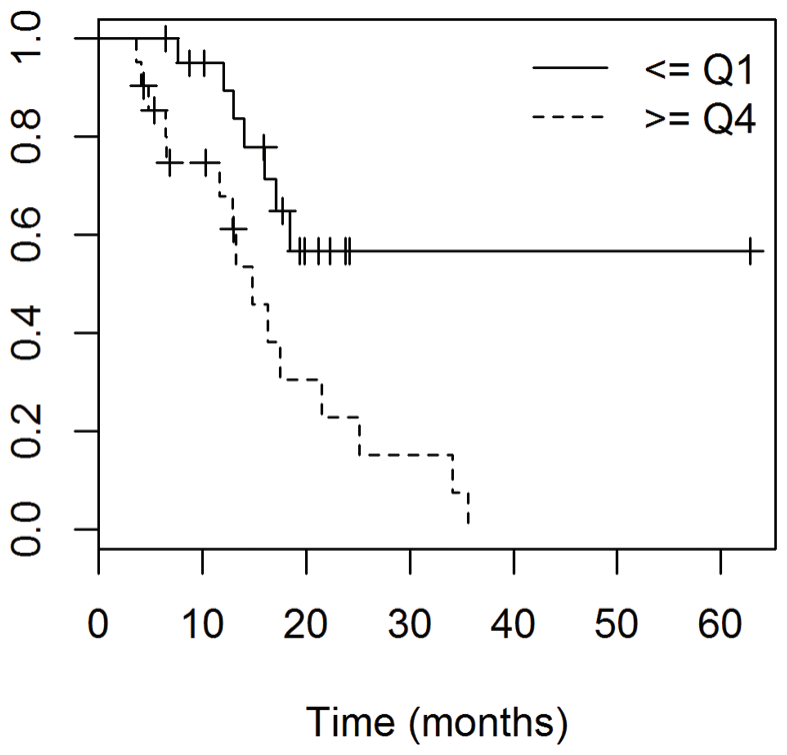
Kaplain-Meier analysis comparing overall survival of patients with the smallest tumors in the bottom quartile (Q1) and patients with the largest tumors in the top quartile (Q4), based on GTV1 volumes. Median OS for patients with the smallest tumors (Q1) was 52.3 months compared to 16.3 months among patients with the largest tumors, *P = 0.006*.

While previous analyses have shown that age and resection status are the primary prognostic factors for survival, tumor size and location are often prognostic in other tumor histologies. To explore the relationship of tumor size defined as the smallest quartile versus the largest quartile [10] and prognosis in GBM, calculated treatment planning volumes were compared to patient outcomes. Clinical variables for the small and large volumes are shown in Table 2. As shown in figure 2, using log rank analysis, patients with the smallest GTV1 tumors (GTV1 in the lowest 25^th^ percentile) had a median OS of 52.3 months compared to 16.3 months among patients with the largest tumors (GTV1 in the highest 25^th^ percentile), *P = 0.006*. Likewise, the median PFS was 12.5 months for patients with the smallest GTV1 tumors and 6.2 months for patients with the largest, *P = 0.008*. On multivariate analysis using tumor volume, age, resection status and location, only tumor volume was statistically significant, *P = 0.*02, Table 2. Similarly, the OS among patients with the smallest PTV1 tumors (lowest 25^th^ percentile) had not reached the median at a follow-up of 17.9 months, compared to 14.8 months in patients with the largest PTV1 tumors (highest 25^th^ percentile), *P = 0.0125*. Patients with the smaller PTV1 tumors had a median PFS of 9.7 months and patients with the larger PTV1 tumors had a median PFS 6.2 months, *P = 0.0186*.

**Table 2:** Tumor Volume.

	Subcategory	Median	Range	Small	Large	HR	SE	p-value
**Number**				21	21			
**Age**								
	<50			6(29%)	2(10%)			
	>50			15(71%)	19(90%)	0.009	0.51	NS
	Median			54	61			
**Resection Status**								
	GTR			11(52%)	3(15%)	0.66	0.88	NS
	STR			6(29%)	13(62%)	0.55	0.82	NS
	Biopsy			4(20%)	5(24%)			
**Location**								
	Non-periventricular			16(84%)	9(45%)			
	Periventricular			3(16%)	11(55%)	0.23	0.60	NS
**Treatment Planning Volumes**								
	GTV1	90.4 cc	2.7–385 cc			1.44	0.63	0.022
	GTV2	28.4 cc	1.6–166 cc					
	PTV1	424.7 cc	76–1124 cc					
	PTV2	340 cc	79–859 cc					

Abbreviations: GTR, gross total resection; STR, sub-total resection; GTV, gross tumor volume; PTV, planning tumor volume.

#### Tumor location

Among the 86 patients, there were 27 (31%) patients with primary tumors categorized as periventricular, and 59 (69%) patients with non-periventricular tumors. Using a log rank analysis, there was a decreased time to progression between the periventricular group and the non-periventricular group, however, there was no significant difference in OS between the groups. The median PFS in patients with periventricular tumors was 5.7 months (95% CI: 5.0–7.9) versus 8.9 months (95% CI: 7.4–11.0) in patients with non-periventricular tumors, *P = 0.005*. The OS was 17.5 months (95% CI: 12.4–26.8) in patients with periventricular tumors, and 23.3 months (95% CI: 16.5–29.0) in patients with non-periventricular tumors, *P = 0.176.* On multivariate analysis using tumor location, age, resection status and tumor volume, PFS for tumor location was no longer statistically significant, Table 3.

**Table 3:** Tumor Location.

	Subcategory	Non-PV	PV	HR	SE	p-value
**Number**		59	27			
**Age**						
	<50	14(24%)	4(15%)			
	>50	45(76%)	23(85%)	0.34	0.31	NS
	Median	56	59			
**Resection Status**						
	GTR	28(47%)	4(15%)	0.45	0.56	NS
	STR	25(42%)	15(56%)	0.54	0.55	NS
	Biopsy	6(10%)	8(30%)			
**Tumor Volume**						
	GTV1	84.7	104.9	0.009	0.003	0.0007

Abbreviations: PV, periventricular: GTR, gross total resection; STR, sub-total resection.

### Use of Bevacizumab at Recurrence

**Figure 3:**
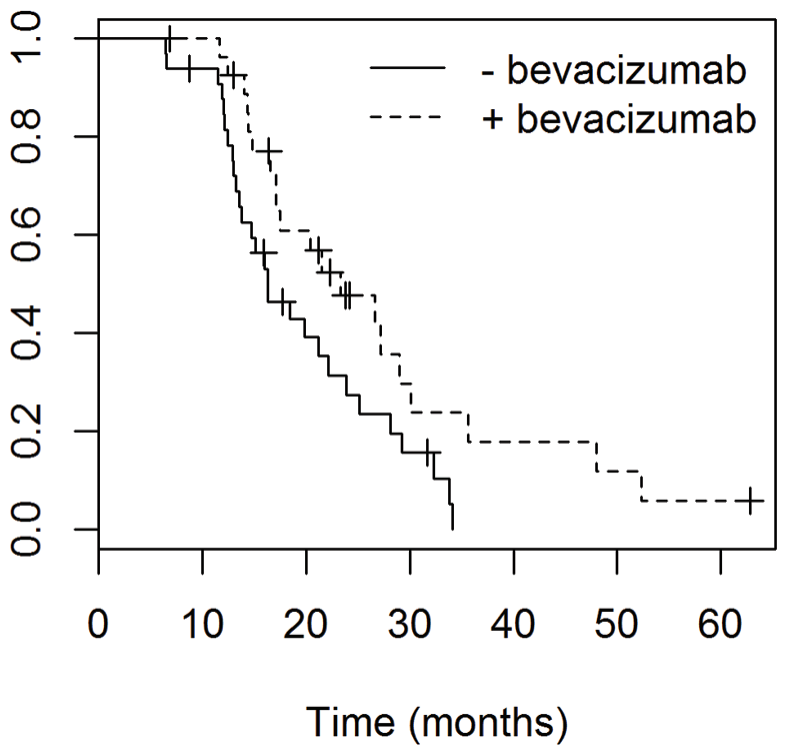
Overall survival of patients who received bevacizumab after progression compared to those who didn’t. Patients receiving bevacizumab had a median OS of 23.3 months (95% CI: 17.1–35.6), compared to 16.3 months (95% CI: 13.8–25.1) in patients who did not receive it, *P = 0.0284.*

Of the 70 patients who progressed, 62 continued to be followed at the NIH. Of those patients, 28 received bevacizumab after their first progression, either as mono-therapy or as part of combination therapy, and 34 did not receive bevacizumab after their first progression. Using log rank analysis, the patients who received bevacizumab had a significant improvement in overall survival. Patients receiving bevacizumab had a median OS of 23.3 months (95% CI: 17.1–35.6), compared to 16.3 months (95% CI: 13.8–25.1) in patients who did not receive it, *P = 0.0284,* Fig. 3. On multivariate analysis using bevacizumab usage, age, resection status and tumor location, bevacizumab usage was statistically significant, *P = 0.*04, Table 4.

**Table 4:** Bevacizumab Usage at Recurrence.

	Subcategory	Yes	No	HR	SE	p-value
**Number**		28	34	0.90	0.44	0.04
**Age**						
	<50	4(14%)	7(21%)			
	>50	24(86%)	27(79%)	0.66	0.44	NS
	Median	59	57			
**Resection Status**						
	GTR	9(32%)	16(47%)	0.85	0.68	NS
	STR	13(46%)	16(47%)	0.57	0.67	NS
	Biopsy	6(21%)	2(6%)			
**Location**						
	Non-periventricular	18(67%)	24(73%)			
	Periventricular	9(33%)	9(27%)	0.011	0.004	0.001

Abbreviations: GTR, gross total resection; STR, sub-total resection.

#### Bevacizumab/Tumor size

**Figure 4:**
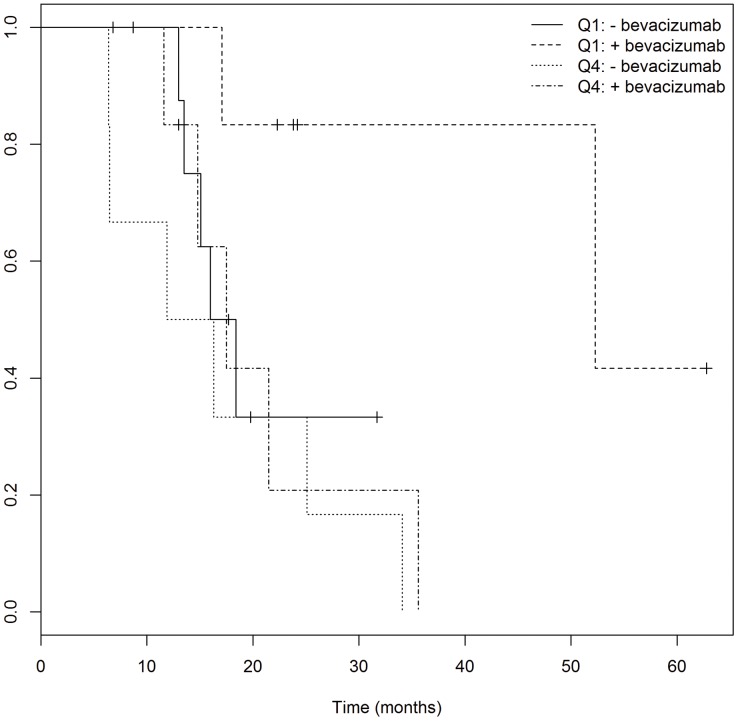
The median OS for the group of patients with the smallest GTV1 tumors who also received bevacizumab (Q1:+bevacizumab) was 52.3 months, compared to 17.2 months for patients with the smallest tumors without bevacizumab (Q1: −bevacizumab), 17.5 months for patients with the largest tumors receiving bevacizumab (Q4: +bevacizumab), and 14.1 months for patients with the largest tumors not receiving bevacizumab (Q4: −bevacizumab).

Moreover, we analyzed the survival outcomes of the patients with the smallest tumors versus those with the largest tumors, and stratified both groups by whether they had received bevacizumab at first recurrence or not (Fig. 4). The survival benefit of bevacizumab was only evident in the group of patients with the smallest tumors (lowest 25^th^ percentile of GTV1 and PTV1). The median OS for the group of patients with the smallest GTV1 tumors who also received bevacizumab was 52.3 months, compared to 17.2 months for patients with the smallest tumors without bevacizumab, 17.5 months for patients with the largest tumors receiving bevacizumab, and 14.1 months for patients with the largest tumors not receiving bevacizumab. This survival benefit remained evident when examining PTV1 volumes: the median OS of patients with the smallest PTV1s who received bevacizumab had not yet reached the median at a follow-up of 26.3 months, whereas the OS for patients with small tumors not receiving bevacizumab, those with large tumors receiving bevacizumab, and those with large tumors not receiving bevacizumab was 18.4, 17.5, and 13.2, respectively.

## Discussion

Herein, we report on a single institution's experience treating 92 consecutive patients with GBM, the majority treated with concurrent radiotherapy and temozolomide, followed by adjuvant temozolomide. Our median PFS and OS of 7.6 and 17.9 months, respectively, are comparable to the EORTC trial results showing a median PFS and OS of 6.9 and 14.6 months, respectively, among patients treated with concurrent and adjuvant TMZ(2). These results validate the efficacy of the EORTC regimen when implemented in routine clinical practice. Two international single center experiences have reported OS of 16.4 and 18.3 months, which are also consistent with our findings [11], [12].

There has been a trend toward improvement in OS among more contemporary GBM studies compared to those treated in the EORTC trial (13–14). Patients from the recent New Approaches to Brain Tumor Therapy (NABTT) single-agent phase II trials, in which all patients received standard RT+TMZ therapy, showed a median OS of 19.6 months [13]. Similarly, another recent phase II trial used its own historical institutional control cohort, which had a median OS of 21.1 months [14]. However, the majority (89%) of those patients received bevacizumab at tumor recurrence, which may have an effect on overall survival. These improvements in survival may be attributed to selection bias in recruitment for phase II trials, or may reflect an improvement in the care of patients with GBM perhaps mirroring the increasing experience using TMZ and a more meticulous monitoring of tumor progression. It is important to consider these improvements in survival when comparing outcomes of phase II studies to historical controls.

It remains obvious that not all patients with GBM have the same prognosis, and that there is a heterogeneous population with varying outcomes. The RTOG-RPA, which was published before the incorporation of TMZ into treatment, reported that certain prognostic factors (age, performance status, tumor histopathology) were stronger prognostic factors than modifications in therapy [15]. The recent validation of these prognostic factors in patient populations treated with TMZ and advanced therapies supports their continued importance relative to changes in therapy [4], [16], [17].

In our study, we report the correlation between volumetric tumor size and progression-free survival and overall survival. The volumetric parameters we examined were the gross tumor volumes using T2 weighted MRI and the initial planning target volume. Our results show that a larger pre-operative, pre-treatment gross tumor volume was associated with a reduction in both PFS and OS, when compared to smaller tumor volume. Importantly, there were an equal percentage of patients that had only a biopsy in each group. Several recent studies have reported an association between GBM tumor size and survival [10], [18]–[20]. One recent study showed a negative impact of pre-operative enhancing tumor, pre-operative necrosis volume, and residual non-enhancing volume prior to radiochemotherapy on outcome [10]. However, they found no association with the preoperative T2 abnormality. A second study concluded that there was no association between pre-operative absolute anatomic lesion volumes and survival, but noted that patients with a large percentage of the T2 lesion containing enhancement and necrosis had a decreased survival [20]. However, when analyzing post-surgery, pre-radiochemotherapy tumor volumes, the same group reported an effect on survival of increasing volumes of all anatomic lesions including T2 and contrast-enhancing lesions [19]. Other studies have reported varying results on whether tumor volume is associated with survival, but were limited by either small sample size or lack of up to date TMZ chemotherapy [21]–[27]. Thus, our data is consistent with previously published data reporting volumetric tumor size as a prognostic marker. In contrast to other studies showing no relationship between T2 abnormality and survival [17], [18], [20], we report that this volume may have prognostic significance, underscoring the importance of an accurate assessment of tumor burden including the most distal tumor cells and edema evident from T2 images. Our data suggest that in the concurrent TMZ era, preoperative tumor volumes on T2 MRI are prognostic of PFS and OS.

A second prognostic factor in our study was the location of the tumor (periventricular vs. non-periventricular). Recent data suggest a role of the cells of the subventricular zone (SVZ) in the GBM stem cell theory. Our study suggests that tumors in contact with the SVZ (periventricular tumors) have a shorter PFS than those patients with non-periventricular tumors, although there was no significant difference in OS. Importantly, there were more patients in the periventricular group that had only a biopsy as their surgical procedure. Two other retrospective studies support an association with tumors involving the SVZ and a decreased survival [28], [29]. However, in the first study, only 58% of patients were evaluated after undergoing a primary resection, and only 27% of patients received TMZ therapy [29]. The second study only evaluated 39 total patients, none of whom received a GTR [28]. Additional studies concluded that patients with tumors adjacent to the SVZ were more likely to be multifocal at diagnosis and to have noncontiguous tumor recurrences [30]. Moreover, those with subependymal spread had decreased survival [31]. Although it remains unknown what underlying biology distinguishes periventricular tumors, our study and others lend support that periventricular tumors may be associated with a decrease in survival even when controlling for extent of resection and use of temozolomide.

Our study also examined the relationship between bevacizumab therapy at tumor recurrence and initial tumor size, showing that among patients receiving bevacizumab, only those with the smaller GTV1 tumors had an increased overall survival of 52.3 months, compared to 17.5 months in those with larger GTV1 tumors (which was comparable to the OS of patients who did not receive bevacizumab at first recurrence). This suggests that bevacizumab might be more effective in smaller tumors, and perhaps the anti-angiogenic effects are less successful once the tumor has reached a certain size. In contrast to our results, one study examined 16 patients with recurrent GBM and noted that hyperperfusion volume was correlated with time to progression, but found no effect of tumor volume at recurrence [32]. Other studies have reported the use of advanced, non-conventional MRI techniques to report response to bevacizumab in recurrent GBM [33]–[35]. One study examining patients with recurrent GBM found that contrast-enhancing volume seen on MRIs taken before bevacizumab initiation were associated with improved PFS but not OS [36]. They reported that a pretreatment ratio of FLAIR to contrast-enhancing volume was associated with PFS and OS [36]. Our study suggests that the tumor volume seen on T2 may be associated with survival. Further studies are needed to clarify whether tumor size can be used as an indicator of potential response to bevacizumab, and whether this can be used to guide treatment decisions.

In conclusion, our data supports previous evidence that preoperative tumor size and tumor location may have prognostic value. Furthermore in our cohort of patients smaller preoperative tumor size is predictive for improved OS when treated with bevacizumab. These findings support current translational research exploring the heterogeneous biology of GBMs and its impact on treatment outcomes.
